# 

**Published:** 1843-01-01

**Authors:** 


### THE MEDICO-CHIRURGICAL REVIEW, AND JOURNAL OF PRACTICAL MEDICINE.

(*NEW SERIES.*)

VOLUME THIRTY-EIGHT,

[1st of OCTOBER, 1842, to 31st of MARCH,]

1843.

VOL. XVIII. of DECENNIAL SERIES.

EDITED

BY JAMES JOHNSON, M.D.

PHYSICIAN EXTRAORDINARY TO THE LATE KING,

AND

HENRY JAMES JOHNSON, Esq.

ASSISTANT SURGEON TO ST. GEORGE'S HOSPITAL,

AND LECTURER ON ANATOMY AT THE SCHOOL OF ST. GEORGE'S HOSPITAL IN KINNERTON STREET.

Surgeon Genl's Office LIBRARY 57478 Washington, D.C.

LONDON:

PUBLISHED BY S. HIGHLEY, 32, FLEET STREET,

*Re-printed in New York, by Mr. Wood.*

1843.

WI
ME 75 T

PRINTED BY F. HAYDEN,
Little College Street, Westminster.

### INDEX.

A

Abscess, hepatic—operation........ 283
Abscess behind the mamma ........ 550
Abscess in the course of the femoral 552
Abscesses in unusual situations, Johnson on........................ 550
Absorption...................... 143
Absorption of pus, D'Arcet on...... 234
Academic information ............ 530
Accident on the Versailles railroad .. 231
Accidents with hard rings .......... 560
Accoucheurs, conduct of .......... 411
Acupuncture in neuralgia .......... 532
Acute hydrocephalus, Bennett on.... 325
African settlements, Madden on the 178
Air, influence of, on pus .......... 498
Air, introduction of, into the veins .. 500
Albuminous tissues, changes in the.. 344
Albuminuria, Alison on............ 405
Aldis's echometer ................. 286
Algeria, diseases of............... 210
Alimentation .................... 447
Alison's outlines of pathology...... 400
Alison on albuminuria ............ 405
Ambi-dexterity ................... 180
Amputation in gangrene .......... 184
Amputation, Mesmeric............ 281
Amputations . ................... 186
Amputations in the Parisian hospitals 209
Anatomists and painters .......... 530
Anatomy of the arteries, Quain's.... 192
Anatomy of sleep, Binn's.......... 465
Ancell's case of malignant tumors .. 24
Aneurism of the aorta ............ 5
Aneurism, false primary .......... 77
Animal development, effects of food on 444
[illegible]nimal development, effects of light on 445
[illegible]terial system, diseases of the...... 453
Animal and vegetable life, Dumas on 522
[illegible]nimals, electrical development in .. 449
Animals, on the feeding of.......... 523
Aorta, aneurism of the............ 5
Aorta, Chevers on diseases of the.... 264
Aorta, vegetations in the .......... 266
Aorta, diminution in the .. ....... 268
Aortic orifice, dilatation of the...... 264
Aqua chalybeata.................... 282
[illegible]quo-capsulitis, Bedford on......... 261
[illegible]quo-capsulitis, symptoms of ...... 261
Aquo-capsulitis, results of.......... 262
A[illegible]ny medical department, Jackson on 95
[illegible]y medical department........... 95
[illegible]my of India, Martin on the...... 95
[illegible]nold on the reflex-function ...... 33
Arrangement of armies, Jackson on.. 95
Art of being ill .................. 468
Arteries, on the tying of .......... 75
Arteries, Quain's anatomy of the.... 192
Arteries, ossification of ............ 453
Artificial penis, Mettauer's case of .. 278
Atlas of anatomy, Cruveilhier's .... 192
Azadirachta Indica................ 540

B.

Baudelocque's obstetrical cases.. ... 506
Beck's case of aneurism of the aorta 5
Bedford on aquo-capsulitis.......... 261
Bell's progress of pharmacy ........ 542
Bengal dispensatory, O'Shaughnessy's 587
Bennett on acute hydrocephalus .... 325
Bennett on the oleum jecoris aselli .. 470
Berberis lycium .................. 539
Bethlem Hospital ....... ........ 202
Binn's anatomy of sleep ... ...... 465
Bipubiotomy, case of.............. 503
Bird on globules in the urine........ 258
Birmingham, vaccination in ........ 556
Bladder, protrusion of, during labour 409
Bladder, Coulson on diseases of the.. 547
Blandin on purulent infection ...... 228
Bleeding, observations on .......... 59
Bleeding from the arm ............ 74
Blindness from sulphuric acid ...... 559
Blood, constitution of the.......... 239
Blood, generation of, in females .... 369
Blood, vitiated state of the ........ 402
Blood, effects of losses of .......... 403
Blood, Hall on determination of, to the head........................ 466
Blood, state of the, in typhoid fever 497
Blood, Lheritier on the ............ 525
Blood, state of the, in diabetes...... 525
Body and mind, preservation of the health of...................... 468
Bombay, Transactions of the Medical Society of .................... 480
Bouillaud in the Chamber of Deputies 208
Bouvier on dog-killing ............ 529
Brain, tubercle of the, in children .. 17
Brain, injuries of the.............. 320
Brain, erethism of the, in infants.... 328
Breech presentations .............. 411
Briquet on quinine in rheumatism .. 530
Brown on Mesmerism ............ 577
Burgess on diseases of the skin...... 475
Burns, ulceration of the duodenum in 30
Burns .......................... 73
Burns, Mutter on cicatrices from.... 273
Burns, Erichsen on .............. 561

C.

Calculi, Scharling on.............. 533
Calculi, discrimination of........... 533
Calculi, physical characters of...... 533
Calculi, classification of............ 534
Calculi, origin and increment of .... 535
Calomel, Gardner on.............. 554
Calotropis Indica................. 540
Cancer of the stomach ............ 486
Cancers, superficial .............. 66
Cancers, observations on .......... 67
Capillaries, diseases of the ........ 457
Carpenter's physiology ............ 440
Carpenter's case of pleurisy ........ 260
Cataract, Morgan on.............. 269
Cataract,congenital,cured by operation 16
Caustics,treatment of ophthalmia with 516
Cayol, Forget's letter to............ 215
Cayol on typhoid fever............. 491
Cazenave on diseases of the skin .... 475
Cells, development of.............. 189
Cerebral tubercle, Dunn's case of.... 21
Cerebral affections, opium in ...... 212
Cervix uteri, elongation of, in sterility 361
Chadwick's sanitary report ........ 373
Chemical analysis, Parnell's ........ 176
Chemistry, Lheritier on pathological 524
Chest, Jeffreys statics of the........ 339
Chevers on diseases of the aorta .... 264
Children, hygiene of .............. 521
Children, tubercle of the brain in.... 17
Chinese treatise on inoculation....... 560
Chlorosis......................... 368
Chomel's elements of pathology.... 452
Chorea, engorgement of the uterus in 365
Christison's dispensatory .......... 112
Churchill on midwifery............ 472
Cicatrices from burns, Mutter on.... 273
Civiale on contractions of the urethra 511
Clark on medical reform .......... 544
Climate of England ............... 202
Clinical medicine, Graves'.......... 434
Clinical instruction ............... 434
Clinical midwifery, Lee's .......... 241
Clinical Surgery, Lisfranc's ........ 56
Clinique chirurgicale, Lisfranc's .... 357
Coculus cordifolius................ 539
Cod liver oil, Bennett on .......... 470
Commentaries on medicine, Crichton's 394
Compression, symptoms of ........ 299
Concussion....................... 294
Concussion, treatment of.......... 297
Congestive pneumonia after operations 556
Consumption, Cooke on............ 478
Conté on the formation of pus...... 527
Contractions of the urethra, Civiale on 511
Cooke on consumption ............ 478
Coptis tecta....................... 539
Coulson on diseases of the bladder .. 547
Counter-irritants ................. 182
Cow-pox, petechial, case of........ 29
Craniotomy ...................... 242
Cranium, fractures of the.......... 294
Cranium, compound fracture of the.. 309
Crichton's commentaries on medicine 394
Crommelinck on lunatic asylums.... 201
Cruveilhier's atlas of anatomy...... 192
Curling on ulceration of duodenum.. 30
Cutaneous diseases, oxyde of zinc in 212
Cyanosis, Walshe's case of ........ 2
Cystoblastema ................... 189

D.

Dalrymple on the placenta.......... 6
D'Arcet on the absorption of pus.... 234
D'Arcet on pus ................... 526
Deafness cured by dental treatment.. 291
Death of the Duke of Orleans ...... 233
Deformities, etiology of............ 517
Delivery, hæmorrhage after ........ 258
Desmarres on caustic in ophthalmia.. 557
Determination of blood to the head, Hull on ...................... 466
Development, arrest of ............ 520
Diagnosis of scabies .............. 531
Dictionnaire des dictionnaires ...... 240
Dipterocarpus lævis .............. 539
Disguised intermittent diseases...... 219
Dispensatory, Bengal,O'Shaughnessy's 537
Dispensatory, Christison's......... 112
Dobson on fortitude under operations 559
Dog-killing Bouvier on ............ 529
Drainage and sewerage ........... 377
Duke of Orleans, death of the...... 233
Dumas on vegetable and animal life.. 522
Dunn's case of cerebral tubercle .... 21
Duodenum, ulceration of the, in burns 30

E.

Echometer, Aldis' ................ 286
Ecthyma ........................ 476
Elements of Physiology, Müller's.... 156
Elements of Physiology, Wagner's .. 156
Elytrotomy....................... 507
Encysted tumor of the neck........ 32
Endocarditis and pericarditis........ 277
England, climate of .............. 202
English, character of the .......... 203
English and foreign spas .......... 565
English spas, Johnson on .......... 565
Engorgement of the uterus ........ 364
Epidemic of typhoid fever, Gibert on 490
Erethism of the brain in infants .... 328
Erichsen on congestive pneumonia .. 556
Erichsen on burns ................ 561
Erysipelas ...................... 184
Etiology of deformities ........... 517
Excision of joints ................ 185
Exercise and inaction.............. 395
Exostosis, non-venereal............ 93
External iliac, ligature of the ...... 76
Extirpation of tumors ............ 79

F.

Falck on veterinary medicine ...... 532

False joints........................ 184
False primary aneurism............ 77
Females, easy regeneration of blood in 369
Femoral, abscess in the course of the 552
Fergusson's surgery .............. 179
Fever, Graves on ................ 435
Fevers, Crichton on .............. 325
Fevers, typhoid, treatment of ...... 396
Fevers, Alison on ................ 405
Fevers, idiopathic ............ ... 406
Fevers, typhoid .................. 407
Fissiparous generation ............ 158
Fistulous sores .................. 83
Floyd on the diseases of Mesopotamia 480
Food, influence of, on plants and insects . ........... ..... .... 520
Food, Truman on ...... ......... 429
Forceps, employment of the........ 241
Forget on the French pharmacopœia 216
Forget's clinique at Strasbourg...... 484
Forget's letter to M. Cayol.......... 215
Fractures, observations on.......... 63
France, lunatic establishments in.... 204
French letter writing .... ........ 528
French pharmacopœia, Forget on the 216
Funeral orations on Larrey ........ 193

G.

Gardner on calomel .............. 554
General pathology, elements of...... 452
Generation by buds ............... 159
Generation, phænomenology of ..... 164
Genital organs, effects of tartar emetic on ......................... 564
Germany, scientific movement in.... 501
Gibert on the epidemic of typhoid fever 490
Glasgow, condition of the poor in.... 377
Globules in the urine, Bird on ...... 258
Gobbi on population .............. 169
Gonorrhœa and syphilis........... 437
Gout, nature of .................. 350
Gout, Liebig's views of............ 352
Gout, treatment of................ 353
Gout, cod-liver oil in.............. 471
Gouty inflammations.............. 401
Gravel, calculus, and gout, Jones on.. 343
Graves' clinical medicine .......... 434
Green on tubercle of brain in children, 17
Gregory's case of petechial cow-pox, 29
Growth of plants ................. 156
Guerin, M., claims of.............. 517
Gums, Koecker on diseases of the . .. 289
Guthrie on injuries of the head...... 293
Guy's Hospital reports ............ 248
Guy's Hospital report ............ 549

H.

Hæmorrhoidal affections, caustic in .. 563
Hæmorrhage after delivery, Lever on, 258
Hæmorrhage from the nose, how to stop ........................ 240
Head, Hull on determination of blood to the ....................... 466
Head, Guthrie on injuries of the ..... 293
Health, influence of food on........ 429
Health, Winslow on the preservation of 468
Heart and arteries, functions of the., 395
Heart, Thompson on malformation of, 27
Heat, evolution of ................ 449
Hepatic abscess—operation ........ 283
Hepatic veins, phlebitis of the ...... 237
Hernia, operation in .............. 531
Hernia, observations on ........... 82
Hip, chronic abscess of the ........ 553
Hip-joint, spontaneous luxation of the 238
Hocken on endo- and pericarditis.... 277
Houston on caustic in hæmorrhoidal affections....................... 563
Hughes on pneumonia ............ 248
Hull on determination of blood to the head............ ............ 466
Human chest, Jeffrey's statics of the, 339
Hydrarthrosis, ioduretted injections in 513
Hydriodate of potash in rheumatism, 515
Hydrocephalus, Bennett on ....... 325
Hydrocephalus, varieties of ........ 325
Hydrocephalus, statistics of ........ 328
Hydrocephalus, frequency of ....... 329
Hydrocephalus, influence of season on 329
Hydrocephalus, morbid anatomy of.. 330
Hydrocephalus, ætiology of ........ 332
Hydrocephalus, treatment of........ 337
Hydrocyanic acid ................ 147
Hydropathy .................... 567
Hygiene, Royer-Collard on ........ 519
Hygiene of children .............. 521
Hysterotomy, results of............ 242

I.

Icones obstetricæ, Moreau's ........ 408
Iliac artery, ligature of, in uterine hæmorrhage ....................... 506
Indian medical boon .............. 571
Indian hemp, O'Shaughnessy on .... 575
Indications of insanity ............ 469
Induction of premature labor, Stolz on 505
Induction of premature labour...... 474
Indurated tumors of the eyelids .... 79
Indurated tissues, resolution of...... 84
Infants, pathological semeiology of .. 174
Infiltration of the labia, sanguineous.. 409
Inflammation, on the treatment of .. 404
Inflammations, rheumatic and gouty, 401
Influence of food on health and disease 429
Injection of dropsical joints.......... 529
Injections, ioduretted in hydrarthrosis 513
Injuries of the head, Guthrie on .... 293
Injuries of the head, symptoms of ... 293
Inoculation, Chinese work on ...... 560
Insane, treatment of the........... 206
Insanity, Crichton on.............. 397
Insanity, definition of ............ 397
Insanity, indications of............ 469
Instinct and reason compared ...... 172
Intermittent diseases, disguised...... 219

Intestines, puncture of, for tympanitis 240
Introduction of air into the veins.... 500
Introductory lectures, Symond's .... 282
Iodine, application of, to the prostate 548
Ioduretted injections in hydrarthrosis, 513
Iris, condition of the, in concussion.. 295
Irish Medical Benevolent Society .... 574
J.
Jackson on military medicine ....... 95
Jeffrey's statics of the chest ...... 339
Jenner, ingratitude of mankind to.... 239
Johnson's cases of abscesses ........ 550
Johnson on English spas .......... 565
Joints, white-swelling of .......... 88
Joints, Velpeau on the injection of .. 529
Jones on gravel, calculus and gout .. 343
K.
King's College Hospital report ...... 549
Koecker on diseases of the gums .... 289
L.
Labia, sanguineous infiltration of the, 409
Labouring classes, condition of the .. 376
Larrey, funeral orations on ........ 193
Laryngitis, cured by operation ...... 10
Lee's clinical midwifery ............ 241
Leeches, application of, to the prostate 547
Leeches, on the application of ...... 58
Leucorrhœa, ætiology of .......... 366
Leucorrhœa produced by café au lait, 367
Lever on hæmorrhage after delivery.. 258
Lheritier, on pathological chemistry.. 524
Ligature of iliac artery in uterine hæmorrhage ..................... 506
Lisfranc's clininal surgery.......... 56
Lisfranc's clinique chirurgicale...... 357
Lithotomy and lithotrity .......... 532
Lithotrity, mode of performing ..... 547
Losses of blood, effects of .......... 403
Lunatic asylum, model of a ........ 206
Lunatic asylums in Germany........ 207
Lunatic asylums, Crommelinck on .. 201
Lunatic asylums in France ......... 204
Lungs, physical diagnosis of diseases of 412
Lupus, treatment of .............. 81
Luxation of the hip-joint, spontaneous 238
Lymphatic system, pathology of the.. 458
M.
Madden on the African settlements.. 178
Malformation of the heart, Thompson on ............................. 27
Malgaigne's statistics of amputations, 209
Malignant pustule ................ 71
Malignant tumors, Ancell's case of .. 42
Mamma, tumors of the............ 357
Mamma, scirrhus of the ........... 358
Mamma, Tanchon on diseases of the.. 516
Mamma, removal of the............ 359
Mamma, abscess behind the........ 550
Man, Neumann on the diseases of ... 452
Man with three testicles ........... 282
Manual of general therapeutics...... 112
Martin on the Indian army ......... 95
Materia medica, Bellingham's ...... 112
Materia medica, Pereira's .......... 112
Medecin, code moral du............ 530
Medical poem..................... 530
Medical reform, Clark on .......... 544
Medical writing, style of .......... 239
Medical department of the army, Jackson on ......................... 95
Medical Benevolent Society of Ireland 574
Medicine, Graves' clinical.......... 434
Medicine, Sir A. Chrichton's commentaries on ....................... 394
Medicines, modus operandi of ...... 116
Medico-Chirurg. Transactions, vol. 25 1
Memoranda on suppuration ........ 526
Menstruation, Boismont on ........ 239
Menstruation, influence of, on vagina, 361
Menstruation ..................... 369
Menstruation, state of ovaries during, 528
Mesmeric amputation ............. 280
Mesmerism, Brown on ............ 577
Mesopotamia, Floyd on the diseases of 480
Metropolis, sewerage of the ........ 577
Metropolis, supply of water to the .. 380
Metropolis, mortality in the ........ 570
Mettauer's case of artificial penis .... 278
Microscope, Paget on the use of the.. 188
Midwifery, Churchill on ........... 472
Midwifery, clinical, Lee's .......... 241
Military surgeon, reminiscences of a.. 199
Moreau's obstetric plates .......... 408
Morgan on cataract ............... 269
Morphology, vegetable ............ 529
Mortality, causes of .............. 375
Mortality in different classes of life .. 383
Mortality in the metropolis ........ 570
Mucous membranes, pathology of ... 462
Müller's physiology .............. 156
Muscular substance, degeneration of, 459
Mütter on cicatrices from burns .... 273
Myotomy and tenotomy ........... 517
Myotomy, extensive, case of........ 568
N.
Neck, encysted tumor of the ....... 32
Necrosis and caries............... 79
Nervous ophthalmia .............. 79
Neuralgia, acupuncture in.......... 532
Newmann on the diseases of man.... 452
Nitrate of silver in ophthalmia ...... 557
Nose, how to stop hæmorrhage from, 240
O.
Obstetric plates, Moreau's.......... 408
Obstetrical cases by Baudelocque.... 506
Oleum jecoris aselli, Bennett on .... 470
Operation, cases of laryngitis cured by 10
Operation, congenital cataract cured by 16
Operation in hernia .............. 531
Operations, removal of dressings after 71
Operations, congestive pneumonia after ............................ 556

Operations, Dobson on fortitude under 559
Ophthalmia, treatment of with caustics 516
Ophthalmia, nitrate of silver in .... 557
Opium in cerebral affections........ 212
Ordinary duration of pregnancy .... 473
Organic and inorganic bodies........ 441
Orleans; death of the Duke of...... 233
O'Shaughnessy's Bengal Dispensatory, 537
O'Shaughnessy on Indian hemp .... 575
Ossification of arteries ............ 453
Out-and-out-ism .................. 240
Outlines of pathology, Alison's...... 400
Ovaries, state of, during menstruation 528
Ovarium, Walne on the removal of the 581
Oxalic acid diathesis .............. 353
Oxalic acid, production of, from uric acid ........................ 354
Oxyde of zinc in cutaneous diseases.. 212

P.

Paget on the symmetry of the body .. 8
Paget on the use of the microscope .. 188
Pancreas, Wilson on disease of the.. 9
Paraplegia ........................ 365
Paralysis, Graves on .............. 437
Parisian hospitals, amputations in the, 209
Parnell's chemical analysis .......... 176
Pathological chemistry, Lheritier on.. 524
Pathology, important discovery in .. 240
Pathology, Alison's outlines of...... 400
Pathology, Chomel's elements of.... 452
Pathology of the venous system .... 455
Pectoralis, abscess beneath the...... 551
Pelviotomy, case of................ 503
Penis, artificial, Mettauer's case of .. 278
Percussion ........................ 414
Pereira's Materia Medica .......... 112
Pereira on hemlock and anise ...... 562
Peri- and endo-carditis, Hocken on.. 277
Petechial cow-pox, case of.......... 29
Pharbitis cœrulea .................. 540
Pharmacy, progress of, Bell's........ 542
Philipps on encysted tumor of the neck 32
Phlebitis of the hepatic veins ....... 237
Phosphatic diathesis .......... .... 355
Phthisis, character of the sputa in ... 207
Physical diagnosis of the diseases of the lungs....................... 412
Physiology of the spinal marrow .... 43
Physiology, Müller's .............. 156
Physiology, Wagner's............... 156
Physiology, Carpenter on .......... 440
Placenta, Dalrymple on the ........ 6
Placental circulation ............... 8
Plants, growth of.................. 157
Plants and insects, influence of food on 520
Pleurisy, Carpenter's case of........ 260
Pleurisy, physical signs of.......... 426
Pneumonia, Hughes on............ 248
Pneumonia, morbid anatomy ....... 249
Pneumonia, chronic, is it rare?...... 249
Pneumonia, general symptoms of.... 249
Pneumonia, physical signs of ....... 250
Pneumonia, treatment of .......... 253
Pneumonia, statistics of............ 254
Pneumonia, physical signs of......... 426
Poisoning, Wilson's cases of ........ 13
Poor-law commissioners, report of the 373
Population, Gobbi on.... .......... 169
Portuguese practice ............... 558
Potash, hydriodate of, in rheumatism 515
Pregnancy, duration of ............. 473
Premature labour, induction of...... 243
Premature labour, induction of...... 474
Premature labour, Stoltz on the induction of........................ 505
Preservation of health, Winslow on.. 468
Progress of pharmacy, Bell's........ 542
Prostate, application of leeches to the 547
Prostate, application of iodine to the 548
Protrusion of the bladder during labour 409
Pruritus of the vulva............... 261
Pulsation in diffused aneurism ...... 77
Purulent infection of the system .... 223
Purulent infection, Blandin on. .... 228
Purulent absorption, D'Arcet on .... 234
Purulent infection ................ 532
Pus, influence of the air on ........ 498
Pus, secondary formations of ...... 317

Q

Quain's anatomy of the arteries .... 192
Quinine in rheumatism ............ 530

R

Reason and instinct compared ...... 172
Reflex-function, Arnold on the...... 33
Reflex-theory of Hall and Müller.... 33
Reflex-theory, opinions of physiologists on ...................... 37
Regimen, Royer-Collard on ........ 519
Remedial treatment, reflections on .. 398
Reminiscences of a military surgeon 199
Removal of the ovarium, Walne on the 581
Report of the poor-law commissioners 373
Reproduction ..................... 450
Respiration, physiology of.......... 340
Rheumatic and gouty inflammations.. 401
Rheumatism, cod-liver oil in........ 471
Rheumatism, hydriodate of potash in 515
Rheumatism, quinine in .......... 530
Ribes, M. reminiscences of ........ 199
Roux's hospital practice .......... 532
Royer-Collard on hygiene.......... 519

S

Sanguineous congestion............ 81
Sanguineous infiltration of the labia.. 409
Sanitary regulations, effects of ...... 389
Sankey on ulcers ................ 564
Sargent on the section of muscles .. 568
Scabies, diagnosis of .............. 531
Scharling on calculi .............. 533
Scientific movement in Germany .... 501
Scirrhus of the mamma............. 358
Secondary formations of pus........ 317

Sedillot on purulent infection ...... 532
Semeiology of infants, Vanier's...... 174
Settlements in Africa, report on the.. 178
Skin, Cazenave on diseases of the.... 475
Sleep, Binn's anatomy of .......... 465
Solvents for the stone............. 536
Spas of England, Johnson on ...... 565
Speculum, use of the.............. 363
Spillan's general therapeutics ...... 112
Spillan's thesaurus medicaminum .. 177
Spinal marrow, physiology of the.... 43
Spontaneous luxation of the hip-joint 238
Sprains ........................ 72
Sputa in phthisis, characters of the.. 207
Squinting, M. Velpeau on.......... 222
Statics of the chest, Jeffrey's........ 339
Sterility, elongation of the cervix in.. 361
Stethoscope, use of, in surgery...... 61
Stolz on the induction of premature labour ...................... 505
Stomach, cancer of the ............ 486
Stone, solvents for the ............ 536
Stone, Mr. Coulson on ............ 547
Strasbourg, Forget's clinique at .... 484
Strasbourg, diseases of ............ 488
Streeter's obstetric plates .......... 408
Stricture of the trachea............ 22
Sulphuric acid, blindness from ...... 559
Suppuration, memoranda on........ 526
Surgery, Lisfranc's clinical ........ 56
Surgery, Fergusson's............... 179
Surgery, Syme's.................. 179
Sydenham society ................ 583
Syme's principles of surgery ...... 179
Symmetry, Paget on .............. 8
Symond's introductory lecture ...... 282
Synochus, treatment of ............ 369
Syphilis, treatment of, without mercury ...................... 399
Syphilis, Graves on............... 437
System, Tessier on purulent infection of the ...................... 223

T

Tanchon on diseases of the mamma.. 516
Tartar emetic, its effects on the genital organs ...................... 564
Teatotalism ...................... 467
Tessier on purulent infection of the system ...................... 223
Testicles, man with three .......... 282
Thalictrum foliosum .............. 538
Thesaurus medicaminum, Spillan's .. 177
Thomson on malformation of the heart 27
Thomson on blindness from sulphuric acid ...................... 559
Trachea, stricture of the .......... 22
Training, effects of............... 521
Transactions of Bombay med. society 480
Trephine on the use of the ........ 310
Truman on food .................. 429
Tubercle of the brain in children.... 17
Tubercle, cerebral, Dunn's case of .. 21
Turkish Arabia, medical topography of 481
Tympanitis, puncture of intestines for 240
Typhoid fevers, treatment of........ 396
Typhoid fever, Gibert on .......... 490
Typhoid fever, Cayol on .......... 491
Typhoid fever, state of the blood in.. 497

U

Ulceration of the duodenum in burns 30
Ulcers, simple.................... 84
Ulcers, external applications to...... 564
Urethra, Civiale on contractions of the 511
Uric acid diathesis ................ 344
Uric acid diathesis, treatment of the.. 347
Uric acid, production of oxalic acid from .......................... 354
Urine, Bird on globules in the ...... 258
Uterine diseases, diagnosis of ...... 363
Uterine hæmorrhage, ligature of iliac artery in ...................... 506
Uterus, engorgements of the........ 364
Uterus, vascular system of the...... 410
Uterus, gravid, pressure from the.... 410
Uterus, mobility of the ............ 361
Uterus, and placenta, connexion between ...................... 410
Uvula, remarks on the ............ 50

V

Vaccination in Birmingham .... ... 556
Vanier's semeiology of infants ...... 174
Varicocele, radical cure of.......... 270
Vascular system of the uterus ...... 410
Vegetable morphology ............ 529
Vegetable and animal life, Dumas on 522
Veins, introduction of air into the .. 500
Velpeau on squinting.............. 222
Velpeau on the injection of joints.... 529
Vena portæ, phlebitis of the........ 237
Venous system, pathology of the .... 455
Ventilation, report on ............ 380
Versailles railroad, accident on the .. 231
Veterinary medicine, importance of.. 532
Vidal on the radical cure of varicocele 270
Vision restored by dental treatment.. 291
Vitalism ........................ 531
Vulva, pruritus of the .... ...... 361

W

Wagner's physiology .............. 156
Walne on the removal of the ovarium 581
Walshe on diseases of the lungs .... 412
Walshe's case of cyanosis .......... 2
White-swelling of joints .......... 88
Wilson on disease of the pancreas .. 9
Wilson on laryngitis .............. 10
Wilson's cases of poisoning ........ 13
Winslow on the preservation of health 468
Worthington on stricture of trachea.. 22
Writer's cramp, operation for ...... 232

### BIBLIOGRAPHICAL RECORD.

1. A Treatise on the Enlarged Tonsil and Elongated Uvula, in connexion with Defects of Voice, Speech, Hearing, and Difficult Deglutition, &c. &c. By James Yearsley, M.R.C.S. &c. Octavo, pp. 83, with Plates. Churchill, Sept. 1842.

---

2. Vital Statistics of Scarborough. By John Dunn, Esq. Surgeon. 4to. London, 1840.

☞ *A good beginning for the local statistics of the "queen of watering places."*

---

3. Galen on the Hand. Translated without Author or Publisher's name. pp. 44.

---

4. Guy's Hospital Reports, No. 15, October 1842. Edited by Drs. Barlow and Babington. Highley, Fleet-street, 1842.

---

5. Pharmaceutical Journal and Transactions. Edited by Jacob Bell. Vol. II. No. 4. October, 1842.

---

6. Natural History of Man. By J. C. Prichard, M.D. No. X. Price 2s. 6d. Bailliere, 1842.

☞ *This valuable work is now come to a successful conclusion.*

---

7. Essays on the Philosophy of Vitality, as contra-distinguished from Chemical and Mechanical Philosophy, and on the Modus Operandi of Remedial Agents. By Martyn Paine, M.D. &c. New York, 1842.

8. La Clinique des Hôpitaux des Enfans, &c. No. 15. Sept. 1842.

---

9. On a Variety of False Aneurism. By Robert Liston, F.R.S.

---

10. Medico-Chirurgical Transactions. Second Series, Vol. VII. 1842.

---

11. Observations regarding Medical Education, in a Letter addressed to the President of the Royal College of Surgeons. By John Simon, Demonstrator of Anatomy in King's College, London. 8vo. pp. 32. Renshaw, 1842.

---

12. An Easy Introduction to Chemistry. By George Sparkes, late of the Madras Civil Service. Duodecimo, pp. 88. Whittaker, 1842.

---

13. Report to Her Majesty's principal Secretary of State for the Home Department from the Poor-law Commissioners, on an Inquiry into the Sanatory Condition of the Labouring Population of Great Britain. With Appendices. 8vo. pp. 457. Printed by Clowes, 1842.

---

14. Retrospect of the Progress of Medicine and Surgery for the Year 1841-2. By E. O. Spooner, Esq. and W. Smart, Esq. &c. Blandford, 1842. Price 3s.

---

15. Elements of Physiology for the use of Students, and with especial reference to the Wants of Practitioners. By RUDOLPH WAGNER, M.D. &c. Translated from the German with Additions, by ROBT. WILLIS, M.D. &c. Part 2, of Nutrition and Secretion, with Wood Engravings. Sherwood and Co. 1842. Price 9s.

16. Food, and its Influence on Health and Disease, &c. By MATTHEW TRUMAN, M.D. Small 8vo. pp. 240. J. Murray, 1842.

17. On the Physiological and Medicinal Properties of Bromine and its Compounds, &c. &c. By R. M. GLOVER, M.D. (From the *Ed. Medical and Surgical Journal.*)

18. Lectures on the Disorders resulting from Defective Nutriment. By G. BUDD, M.D. F.R.S. &c. (From the *Medical Gazette.*)

19. A System of Practical Surgery. By WILLIAM FERGUSSON, F.R.S.E. Professor of Surgery in King's College, &c. &c. 8vo. pp. 596. Churchill, 1842.

20. The Anatomy of Sleep; or Art of Procuring sound and refreshing Slumber at Will. By ED. BINNS, M.D. Octavo, pp. 392. Churchill, 1842.

21. An Account of Askern and its Mineral Springs, &c. &c. By EDWIN LANKESTER, M.D. F.L.S. 8vo. pp. 151. Churchill, 1842.

22. Clinical Midwifery: with Histories of 400 Cases of Difficult Labour. By ROBERT LEE, M.D. F.R.S. Fellow of the Royal College of Physicians, &c. Octavo, pp. 224. Churchill, 1842.

23. On Injuries of the Head affecting the Brain. By G. J. GUTHRIE, F.R.S. Surgeon to the Westminster Hospital, &c. 4to. pp. 155. Churchill, 1842.

24. Observations on some Points in the Anatomy, Physiology, and Pathology of the Blood. By T. WHARTON JONES, F.R.S. Lecturer on Anatomy, &c. at the Charing Cross School. Octavo, pp. 24. Churchill, 1842.

25. Report of the Results obtained by the use of the Microscope in the Study of Human Anatomy and Physiology. By JAMES PAGET, Demonstrator of Morbid Anatomy, Bartholomew's Hospital. 8vo. pp. 51. Churchill, 1842.

26. A History of British Birds. By W. YARRELL, F.L.S. for August, September, and October, 1842. Van Voorst, 1842.

27. Annals of Chemistry and Practical Pharmacy. No. 3. Longman and Co. Oct. 1842.

28. The Medical Times. Quarterly Number up to October, 1842. *In Exchange.*

29. Manual of Diseases of the Skin. From the French of MM. Cazenave and Schedel. With Notes and Additions. By THOMAS H. BURGESS, M.D. Surgeon to the Blenheim Street Dispensary. Octavo, pp. 320. Renshaw, 1842.

30. The Pocket Formulary; and Synopsis of the British and Foreign Pharmacopœias, comprising sundry Standard and Approved Formulæ, &c. &c. By HENRY BEASLEY. Second Edition. Duodecimo, pp. 284. Sherwood and Co. 1842.

31. The Second Supplement: completing the Seventh Edition of Dr. Turner's Chemistry. By JUSTUS LEIBIG, M.D. and WILLIAM GREGORY, M.D. Price 3s. Taylor and Walton, Nov. 1842.

32. Observations on the Admission of Medical Pupils to the Wards of Bethlem Hospital, for the purpose of Studying Mental Diseases. Third Edition revised. By JOHN WEBSTER, M.D. one of the Committee.

☞ *This edition is enriched by many valuable observations and notes made during a recent visit to several Hospitals for the Insane on the Continent.*

33. Dr. Symonds' Introductory Lecture delivered at the Bristol Medical School.

34. A Dictionary of Practical Medicine. By JAMES COPLAND, M.D. F.R.S. &c. &c. Part VIII. Articles—Kidneys, and Lungs. Longman and Co. Nov. 1842.

35. Observations on the Expediency of Abolishing Mechanical Restraint in the Treatment of the Insane in Lunatic Asylums. By JOHN CRAWFORD, M.D. 8vo. sewed, pp. 36. Highley, London, 1842.

36. Observations and Facts relating to the Deaf and Dumb, &c. By W. WRIGHT, Lecturer on the Ear. 8vo. pp. 16. Strange, Paternoster-row, 1842.

---

37. Atlas of the Anatomy of the Human Body. By J. CRUVEILHIER, M.D. Parts 14 and 15. Bailliere, Regent Street.

---

38. On the Preservation of the Health of Body and Mind. By FORBES WINSLOW, M.R.C.S. &c. &c. 8vo. pp. 202. Renshaw, Strand, 1842.

☞ *In our next.*

---

39. Chemistry of Animal Bodies. By THOMAS THOMSON, M.D. Regius Professor in the University of Glasgow, &c. &c. Pp. 702. Longman and Co.

---

40. The Pharmaceutical Journal and Transactions. Vol. 2, No. 6. Dec. 1842.

---

41. Praxeos-Medicæ Universæ Præcepta. Auctore JOSEPHE FRANK. Leipsiæ, 1841. Longman and Co. London.

---

42. Thesaurus Medicaminum; or the Medical Prescriber's Vade Mecum, &c. By D. SPILLAN, M.D. Duodecimo, pp. 154. Bailliere, Regent-street, 1842.

☞ *An excellent little pocket companion for the active practitioner.*

---

43. On Gravel, Calculus, and Gout; chiefly an Application of Liebig's Physiology to the Prevention and Cure of these Diseases. By H. BENCE JONES, M.A. Cantab. &c. Octavo, pp. 142. Taylor and Walton, London, Dec. 1842.

---

44. The Naturalist's Library; conducted by Sir W. JARDINE, Bt. &c. Introduction to the Mammalia, by Lieut.-Col. Charles Hamilton Smith, &c. Vol. XIII. Lizars. Edinburgh—Highley, London, Dec. 1842,

45. A Practical Treatise on Pulmonary Consumption, its Pathology, Diagnosis and Treatment, &c. By FRANCIS COOK, M.D. M.R.C.S. Physician to the South General Dispensary. Octavo, pp. 129. Churchill, London, Dec. 1842.

---

46. Retrospective Address delivered at the Tenth Anniversary Meeting of the Provincial Medical and Surgical Association. By JAMES BLACK, M.D. Physician to the Manchester Union Hospital.

---

47. A Practical Treatise on the Human Teeth, &c. With Plates. By WILLIAM ROBERTSON. Third Edition. J. Churchill, Dec. 1842.

---

48. Physician for Ships; containing Medical Advice for Seamen and other persons at Sea, on the Treatment of Diseases, and on the Preservation of Health in Sickly Climates. By USHER PARSONS, M.D. late Surgeon in the United States' Navy, &c. Boston, 1842. Duodecimo, pp. 218.

---

49. Lectures on Animal Physiology, or the Physical Condition of Man, in relation to Life, Health, and Disease, &c. By B. T. LOWE, of St. Bartholomew's Medical School. Small 8vo. pp. 101. Price 2s. 6d. London, Simpkin and Marshall, 1842.

---

50. The Physical Diagnosis of Diseases of the Lungs, &c. By WALTER HAYLE WALSHE, M.D. 8vo. pp. 307. Taylor and Walton, London, Dec. 1842.

---

51. Recommendations for filling-up the Register of Cases, agreed to at the Annual Meeting of the Association of Medical Officers of Hospitals for the Insane, held in Lancaster, June 1842.

☞ *We would strongly recommend this form of register, with the queries on which it is founded. It may be procured, at cost price, from Dr. Hitch, Asylum, Gloucester.*

### TO CORRESPONDENTS.

Several Communications are under consideration—among others, the Cases from the Note-Book of Mr. COOKE, of America; but we wish it to borne in mind that we are unable to insert Original Communications, except in the EXTRA-LIMITES, and at the expense of the Author. It will be better, therefore, for such Authors to transmit their Papers to some of the various Journals that have Departments for those purposes.

